# Characterization of Pairwise Correlations from Multiple Quantum Correlated Beams Generated from Cascaded Four-Wave Mixing Processes

**DOI:** 10.1038/srep40410

**Published:** 2017-01-10

**Authors:** Hailong Wang, Leiming Cao, Jietai Jing

**Affiliations:** 1 State Key Laboratory of Precision Spectroscopy, School of Physics and Materials Science, East China Normal University, Shanghai 200062, People’s Republic of China; 2 Collaborative Innovation Center of Extreme Optics, Shanxi University, Taiyuan, Shanxi 030006, People’s Republic of China

## Abstract

We theoretically characterize the performance of the pairwise correlations (PCs) from multiple quantum correlated beams based on the cascaded four-wave mixing (FWM) processes. The presence of the PCs with quantum corre- lation in these systems can be verified by calculating the degree of intensity difference squeezing for any pair of all the output fields. The quantum correlation characteristics of all the PCs under different cascaded schemes are also discussed in detail and the repulsion effect between PCs in these cascaded FWM processes is theoretically predicted. Our results open the way for the classification and application of quantum states generated from the cascaded FWM processes.

Quantum correlation shared between multiple quantum correlated beams is important for fundamental quantum mechanics^1^ and significant applications in quantum information technologies^2^. The relationship between the quantum correlation shared by the multiple quantum correlated beams and the pairwise correlations (PCs) of the multiple beams remains an open question. For example, ref. 3 discusses the trade-off between A’s correlation with B and its correlation with C in a three qubits (A, B and C) system; ref. 4 reviews the properties of the PCs in many-body systems; refs 5, 6 and ref. 7 give the classification of three-qubit correlation and four-qubit correlation respectively which both involve the consideration of PCs. ref. 8 have formalized and extended the operational classification and quantification of multipartite correlated states related to the PCs. Therefore, the characterization of PCs existed in the multiple quantum correlated beams is worth investigating for both the classification and application of quantum states.

Four-wave mixing (FWM) process in a hot rubidium (Rb) vapor^9^,^10^,^11^,^12^,^13^,^14^,^15^,^16^,^17^,^18^,^19^,^20^,^21^,^22^,^23^,^24^ has several advantages of practical implementations, e.g., no need of cavity due to strong nonlinearity of the system, natural spatial separation of the generated non-classical beams, etc. Our group has experimentally demonstrated the generation of strong quantum correlation between the three bright beams from a cascaded FWM process^25^. Under that experimental condition, there doesn’t exist any quantum correlation between any two of the three beams, i. e., no PC with quantum correlation has been shown in our previous work. Therefore, the dependence of the PCs on the system operating condition of the cascaded FWM processes is very interesting and worth studying. In this letter, based on two different cascaded FWM processes, i. e., asymmetrical cascaded scheme and symmetrical cascaded scheme, we theoretically characterize the performance of the PCs of the multiple quantum correlated beams and analyze their dependences on the system intensity gains *G*_*k*_ (*k* = 1, 2). The theoretical predictions can give a rough estimation of the obtained experimental results.

## Results

### Single FWM scheme

**Figure 1:**
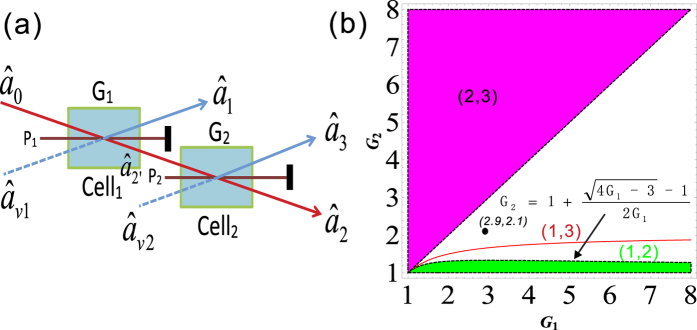
(**a**) Asymmetrical cascaded scheme. 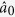 is coherent input signal, 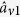 and 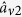 are vacuum inputs, *G*_1_ and *G*_2_ are the power gains of cell_1_ and cell_2_ respectively. 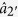 is the output signal beam from the first FWM, 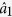, 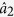 and 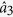 are the triple output beams. P_1_ and P_2_ are the pump beams for the Cell_1_ and Cell_2_ respectively. (**b**) The region plot of Eq. (8), Eq. (9) and Eq. (10). The green region (1, 2) is the region of 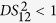, the red line (1, 3) is the region of 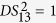 (*G*_2_ = 2 − 1/*G*_1_), the magenta region (2, 3) is the region of 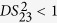. The black point (2.9, 2.1) is the experimental gain point. The blank region is the region in which 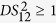, 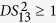 and 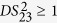. In all region, 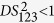.

Firstly, we give a simple description of the single FWM scheme. FWM is a nonlinear process in which two pump photons can convert to one signal photon and one idler photon, or vice versa. In the cell_1_ of Fig. 1(a), an intense pump beam and a much weaker signal beam are crossed in the center of the Rb vapor cell with a slight angle. Then the signal beam is amplified as 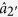 and a new beam called idler beam is generated as 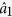 on the other side of the pump beam at the same time. The signal beam and idler beam have different frequencies. The input-output relation of the single FWM scheme shown in Fig. 1(a) is given by


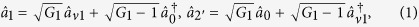


where *G*_1_ is the power gain of the FWM process. 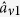 is the vacuum input and 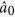 is the coherent input. Following the expressions of the creation and annihilation operators, the optical intensities (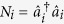 (*i* = 1, 2’)) for the beams 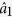 and 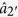 can be given by


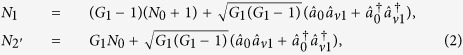


where 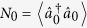. Then the PC for the two beams 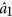 and 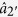 can be quantified by the degree of intensity difference squeezing (*DS*), i. e., the ratio of the variance of the correlated beams to the variance at the standard quantum limit (SQL)^26^


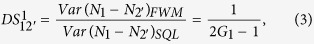


here 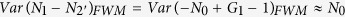 and 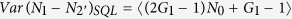

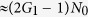
. Here the superscript and subscript for the 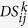 represent the *k*th (*k* = 1, 2, 3) scheme (we have three schemes throughout the whole discussion, i. e., the single FWM scheme (1), the asymmetrical cascaded scheme (2) and the symmetrical cascaded scheme (3), the *i*th (*i* = 1, 2, 3) beam and the *j*th (*j* = 2′, 2, 3, 4) beam in the scheme. *Var (A*) = 〈*A*^2^〉 − 〈*A*〉^2^ denotes the variance of A. 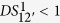 demonstrates the presence of PC with quantum correlation between the two beams from the FWM process. Since *G*_1_ is always larger than 1, the PC with quantum correlation of the two beams can be easily obtained in the experiment. However, the DSs of the single beams 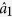 and 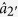 are given by


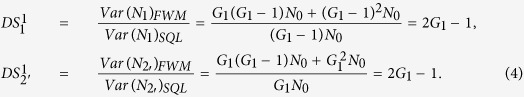


This corresponds to a linear increase in the noise on both the signal and idler beams as gain is increased. Thus the beams 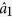 and 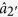 in the FWM process are both in thermal states.

### Asymmetrical cascaded FWM scheme

**Figure 2:**
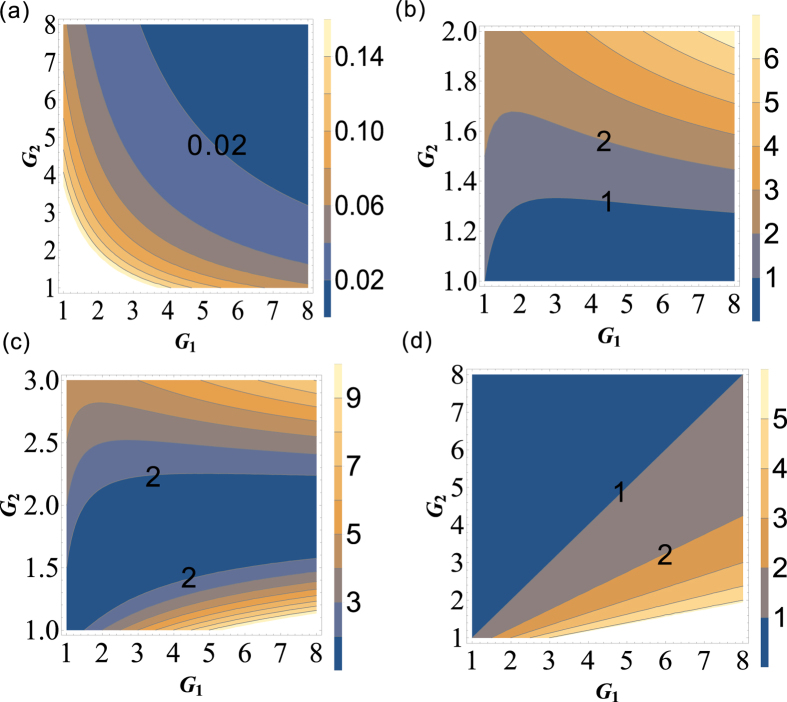
The contour plot of (**a**) 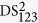; (**b**) 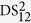; (**c**) 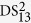 and (**d**) 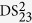.

Secondly, compared to the above mentioned single FWM scheme, here we construct the asymmetrical cascaded scheme in Fig. 1(a). We take the signal beam from the first FWM process (cell_1_) as the seed for the second FWM process (cell_2_) in Fig. 1(a)^25^. 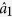, 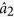 and 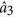 are three newly-generated beams in the output stage of the cascaded processes. In our previous work^25^, we have shown the generation of strong quantum correlation between the three bright beams but not the PCs with quantum correlation for any pair of the three beams. Here we will study all the PCs of the triple beams 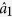, 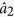 and 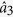 in Fig. 1(a) and look for the possibilities for the existence of PCs with quantum correlation. The input-output relation of the asymmetrical cascaded scheme in Fig. 1(a) can be written as


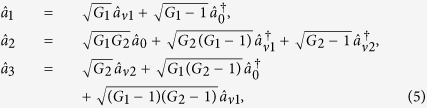


where *G*_1_ and *G*_2_ are the power gains for the cell_1_ and cell_2_ respectively. The optical intensities (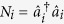 (*i* = 1, 2 and 3)) for the individual beams 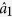, 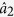 and 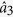 can be given by


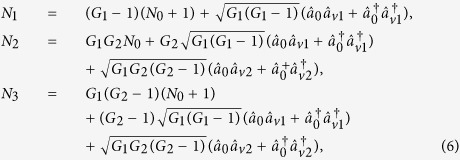


where 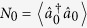. Here the second-order vacuum terms are omitted. It should be noted that the *DS* of the triple beams (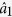, 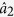 and 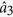) is given by


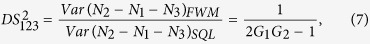


where *G*_1_ and *G*_2_ are the power gains for the two FWM processes. Compared with Eq. (3), Eq. (7) means that the cascaded FWM process can enhance the quantum correlation of the system. The symmetrical dependence of the 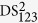 on the gains is shown in Fig. 2(a) and can be enhanced with the increasing of the gains G_1_ and G_2_. The quantum correlation shared by the triple beams is present if *G*_1_*G*_2_ > 1, i. e., *G*_1_ > 1 or *G*_2_ > 1.

Next we analyze all the possible PCs using the *DS* criterion. PC between 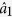 and 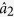 can be quantified by


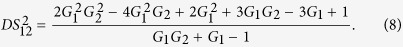


Eq. (8) will be reduced to 2*G*_2_ − 1 and 1/(2*G*_1_ − 1) when we set *G*_1_ = 1 and *G*_2_ = 1 respectively, corresponding to the cases of Eq. (4) and Eq. (3) respectively. These phenomena can be understood as follows. When we set *G*_1_ = 1, the PC between the thermal states 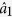 and 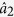 translates into the one between the vacuum state 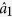 and the thermal state 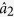, i. e., 
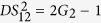
. When we set *G*_2_ = 1, the PC between the thermal states 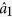 and 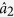 translates into the one between the twin beams from the first FWM process, i. e., 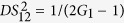. The region in which 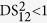, i. e., there exists quantum correlation between beams 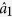 and 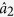, is shown in green denoted as (1, 2) in Fig. 1(b). The value of *G*_2_ on the boundary (see the boundary given by 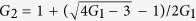 in Fig. 1(b)) of that region reaches its maximal value of 1.33 when *G*_1_ = 3 and it decreases when *G*_1_ > 3 and will eventually reaches at the value of 1. To clearly see how the 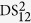 depends on the gains *G*_1_ and *G*_2_, the contour plot of it is shown in Fig. 2(b). The larger G_1_ and smaller G_2_ are preferred for achieving 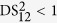. The study of 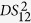 presented above is actually the question of how to preserve the quantum correlation between beams 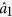 and 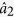 under the introduction of a second FWM which brings the deterioration effect to the quantum correlation by the quantum amplification of one of the beams (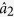). The results shown in Figs 1(b) and 2(b) actually shows the value of *G*_2_ on the boundary below which the quantum correlation can always be preserved as the value of *G*_1_ increases. That is to say, in the low gain regime (*G*_1_ < 3), the stronger the quantum correlation between beams 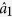 and 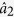 is, the more robust to the deterioration effect from the quantum amplification of the second FWM it becomes. More interestingly, in the high gain regime (*G*_1_ > 3), the stronger the quantum correlation between beams 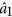 and 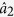 is, the more fragile to the deterioration effect from the quantum amplification of the second FWM it becomes.

PC between 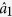 and 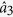 can be quantified by


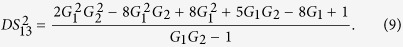


Eq. (9) is equal to 1 when *G*_2_ = 2 − 1/*G*_1_, meaning that the quantum fluctuation of intensity difference of two thermal states can be equal to the one of two coherent states with equal powers. Except that, 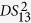 is always larger than 1, i. e., there is no quantum correlation between beams 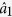 and 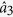. The region in which 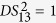 (*G*_2_ = 2 − 1/*G*_1_) is shown as the red line denoted as (1, 3) in Fig. 1(b). The contour plot of 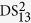 is also shown in Fig. 2(c) in which the value of all the region is more than or equal to 1 for any G_1_, G_2_ > 1. In this sense, there isn’t any quantum correlation between beams 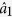 and 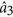 for any value of *G*_1_ and *G*_2_ since 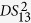 is always more than or equal to 1.

PC between 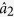 and 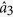 can be quantified by


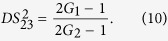


The simplified results, i. e., 1/(2*G*_2_ − 1) and 2*G*_1_ − 1 for Eq. (10) can be obtained when *G*_1_ and *G*_2_ are set to equal to 1 respectively, corresponding to the cases of Eq. (3) and Eq. (4) respectively. This is because when we set *G*_1_ = 1, the PC between the thermal states 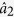 and 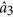 translates into the one between the twin beams from the second FWM process, i. e., 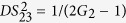. When we set *G*_2_ = 1, the PC between the thermal states 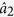 and 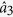 translates into the one between the thermal state 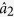 and the vacuum state 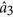, i. e., 
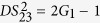
. The region in which 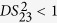 (*G*_1_ < *G*_2_) is shown in magenta denoted as (2, 3) in Fig. 1(b), meanwhile, the contour plot of 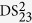 is also shown in Fig. 2(d) in which the region of *G*_1_ < *G*_2_ gives 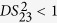. Therefore, the PC with quantum correlation between beams 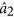 and 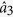 will be present for any *G*_1_ less than *G*_2_. This is not difficult to figure out if one looks at the functional form of Eq. (10). As we all know, in order to generate strong quantum correlation from FWM process, the shot noise limited seed beam, such as coherent state or vacuum state, is always preferred. From this point of view, the analysis presented above actually answers the question of how to produce quantum correlation with the seeding of a thermal state. Figs 1(b) and 2(d) actually gives the answer that the quantum correlation will be produced as long as the FWM gain for producing the quantum correlation is larger than the FWM gain for the thermal state generation. In such region (*G*_1_ < *G*_2_), the existence of quantum correlation between beams 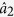 and 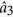 eliminates the possibility of the one between beams 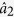 and 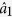. In other words, beam 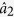 can’t be simultaneously quantum correlated with beams 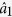 and 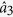. In this sense, we could call this phenomena as repulsion effect of quantum correlation between the PCs in this cascaded FWM process. It can be explained as follows. The repulsion effect is actually the result of the competition between the correlation mechanism and decorrelation mechanism. As shown in Fig. 1(a), firstly, for the PC between beams 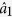 and 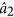, obviously, cell_1_ will provide the correlation between them and cell_2_ will destroy their quantum correlation by adding extra vacuum noise, thus cell_1_ and cell_2_ can be viewed as the correlation mechanism provider and decorrelation mechanism provider respectively, thus the larger G_1_ and smaller G_2_ are preferred for the PC between beams 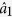 and 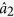. Secondly, for the case of the PC between beams 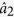 and 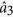, cell_1_ will generate a thermal state 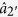 which will destroy their quantum correlation by adding extra vacuum noise into the system while the cell_2_ will make them quantum correlated through the FWM process. In this case, cell_1_ and cell_2_ can be viewed as the decorrelation mechanism provider and correlation mechanism provider respectively, thus the smaller G_1_ and larger G_2_ are preferred for the PC between beams 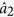 and 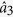. Finally, the complete opposite dependence of the PC between beams 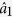 and 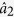 and the PC between beams 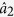 and 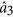 on the gains leads to the repulsion effect between the PCs of certain pairs. In the blank region of Fig. 1(b), all the PCs with quantum correlation are absent since 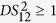, 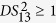 and 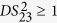, however, the quantum correlation between the triple beams is still present.

**Figure 3:**
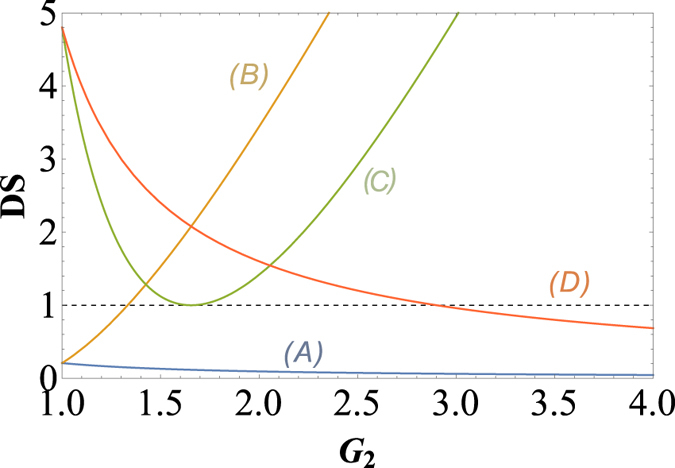
The dependence of (*A*) 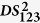; (B) 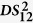; (*C*) 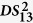 and (*D*) 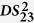 on the gain G_2_ when G_1_ = 2.9 (cell_1_ gain in the experiment). The black dashed line: SQL.

In order to give a summary of the theoretical predictions of Figs 1(b) and 2. We plot the dependence of (A) 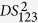; (B) 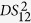; (C) 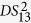 and (D) 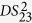 on the gain G_2_ when G_1_ = 2.9 (cell_1_ gain in the experiment) in Fig. 3. 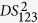 (trace A) can be enhanced with the increasing of *G*_2_ which is consistent with Fig. 2(a), the value of 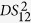 (trace B) will be larger than 1 as long as *G*_2_ > 1.33 which is consistent with the boundary of 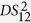 in Fig. 1(b), 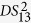 (trace C) will approach the SQL only G_2_ = 1.66 which is consistent with *G*_2_ = 2 − 1/*G*_1_ in Fig. 1(b), the value of 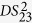 (trace D) will be smaller than 1 as long as *G*_2_ > 2.9 which is consistent with Figs 1(b) and 2(d).

**Figure 4:**
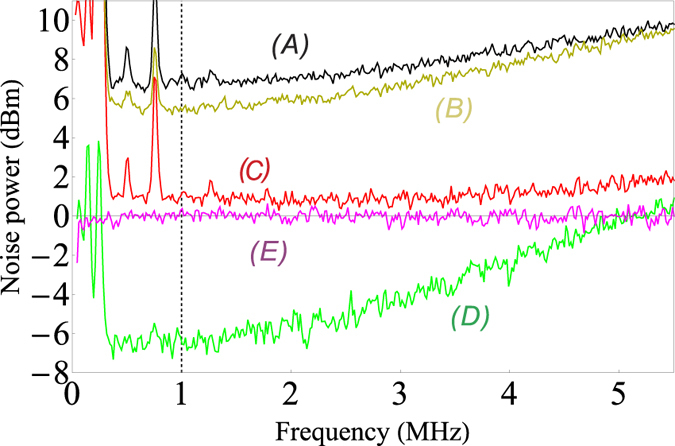
Experimentally measured (*A*) 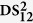; (*B*) 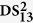; (*C*) 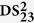; (*D*) 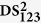 and (*E*) the corresponding SQLs of the traces *A* ~ *D* in the asymmetrical cascaded scheme. The vertical dashed line: 1 MHz.

To verify these theoretical predictions, we apply them to the experimental results. The measured results are shown in Fig. 4, the traces *A, B, C* and *D* are the measured DSs between 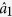 and 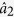, 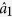 and 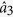, 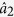 and 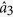 and the triple beams respectively, the trace *E* is the corresponding normalized SQLs for traces *A* ~ *D* (See the methods). The experimental results show 10Log(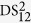) = 7.0 ± 0.2 dB, 10Log(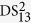) = 5.5 ± 0.1 dB, 10Log(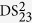) = 1.0 ± 0.2 dB and 10Log(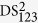) = −6.7 ± 0.4 dB at 1 MHz where the maximal degree of squeezing can be considered as the best choice to reflect the quantum properties of the system because there exist huge classical noise peaks at lower frequencies from the laser, the bandwidth limitation of the photodetector and even the bandwidth limitation of the squeezing generation. As we can see from Fig. 4, the noise power of the three beams increases quickly as the frequency increases. It also increases faster than the one of the two beams. We can understand this results as follows. Although the probe and idler beams in the single FWM scheme are generated almost simultaneously, there are still some time delay between them during their propagation through the cell^27^. This difference limits the squeezing bandwidth to some extent. This time delay induced squeezing bandwidth becomes narrower in the case of asymmetrical cascaded FWM scheme due to two of the three beams experiencing additional time delay in the second vapor cell. The faster increasing of the noise power of the three beams than the one of the two beams is due to that the number of beams of the three beams related to the time delay is more than the one of the two beams. For the experimental gains *G*_1_ ≈ 2.9 and *G*_2_ ≈ 2.1, our theoretical predictions give 10Log(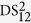) = 5.9 dB, 10Log(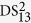) = 2.2 dB, 10Log(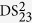) = 1.8 dB and 10Log(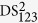) = −10.5 dB in which the positive and negative values represent antisqueezing and squeezing respectively. As we can see, although these theoretical predictions do not perfectly agree with the experimental results at 1 MHz, they still give a rough estimation of the relationship between the obtained experimental noise power traces.

### Symmetrical cascaded FWM scheme

**Figure 5:**
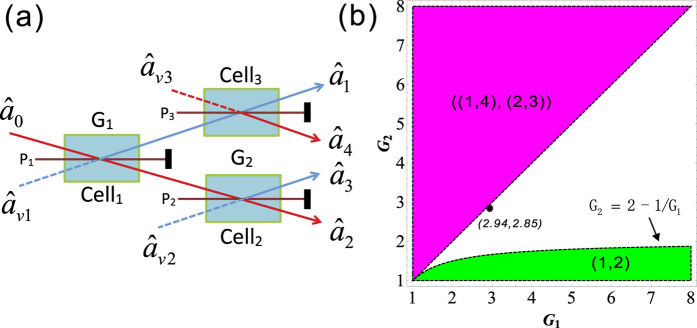
(**a**) Symmetrical cascaded scheme. 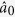 is coherent input signal, 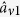, 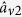 and 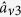 are vacuum inputs, *G*_1_ and *G*_2_ are the power gains of cell_1_ and cell_2_ (cell_3_) respectively. 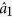, 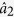, 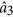 and 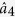 are the output beams. P_1_, P_2_ and P_3_ are the pump beams for the Cell_1_, Cell_2_ and Cell_3_ respectively. (**b**) The region plot of Eq. (14), Eq. (15). The green region (1, 2) is the region of 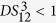, the magenta region ((1, 4), (2, 3)) is the region of 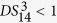 and 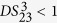. The black point (2.94, 2.85) is the experimental gain point. The blank region is the region of 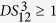, 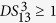, 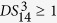, 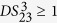, 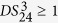 and 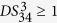, meaning that there is no PC with quantum correlation for any pair of the quadruple beams. In all region, 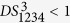.

**Figure 6:**
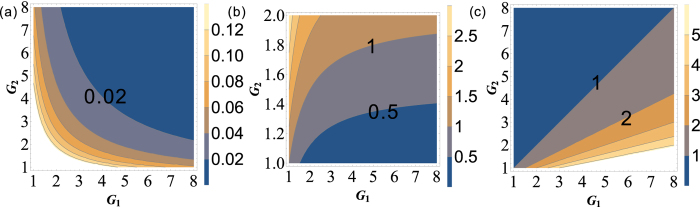
The contour plot (**a**) 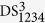; (**b**) 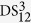 and (**c**) 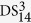 and 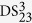.

Finally, we construct the following symmetrical cascaded scheme as shown in Fig. 5(a). We take the signal beam from the first FWM process (cell_1_) as the seed for the second FWM process (cell_2_) and the idler beam as the seed for the third FWM process (cell_3_) in Fig. 5(a). 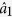, 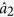, 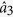 and 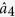 are the quadruple newly-generated beams in the output stage of the cascaded processes. We will also study all the PCs of the quadruple beams 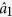, 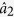, 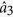 and 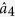 in Fig. 5(a) and look for the possibilities for existence of the PCs with quantum correlation. The input-output relation of the symmetrical cascaded scheme in Fig. 5(a) can be written as


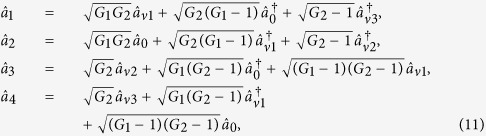


where *G*_1_, *G*_2_ are the power gains of cell_1_, cell_2_ (cell_3_) respectively. Here we assume that the two FWM processes in the cell_2_ and cell_3_ have the same power gains for simplicity. The optical intensities (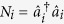 (*i* = 1, 2, 3 and 4)) for the individual beams 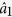, 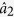, 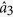 and 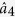 can be given by


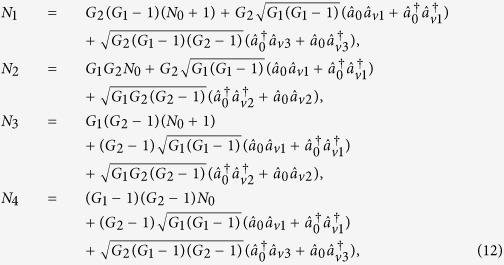


where 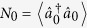. Here the second-order vacuum terms are omitted. It should be noted that the *DS* of the quadruple beams (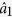, 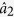, 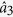 and 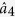) is given by


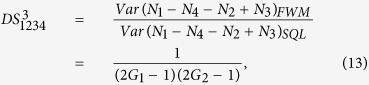


compared with Eq. (7), this cascaded scheme has also enhanced the quantum correlation of the system. The symmetrical dependence of the 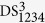 on the gains is shown in Fig. 6(a) and it can be enhanced with the increasing of the gains *G*_1_ and *G*_2_, similar to the case of 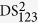. The quantum correlation shared by the quadruple beams is present if (2*G*_1_ − 1)(2*G*_2_ − 1) > 1, i. e., *G*_1_ > 1 or *G*_2_ > 1. Next let us analyze all the possible PCs in this scheme and the triple correlations can also be obtained from the expressions of the PCs in the asymmetrical cascaded FWM scheme, for example, the DS for the triple beams (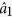, 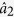 and 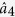) can be expressed as 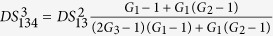. Therefore, we only focus on the PCs. PC between 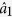 and 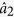 can be quantified by


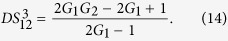


The region in which 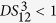 is shown in green denoted as (1, 2) in Fig. 5(b), the contour plot of the dependence of 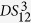 on the gains *G*_1_ and *G*_2_ is shown in Fig. 6(b). With the increasing of *G*_1_, the value of *G*_2_ on the boundary always increases and eventually saturates at the value of 2 (see the boundary given by *G*_2_ = 2 − 1/*G*_1_ in Fig. 5(b)). This is different from the asymmetrical scheme discussed above, where the value of *G*_2_ on the boundary finally reaches 1. This is because here beams 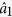 and 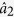 from the first FWM process experience the same amount of amplification in the second and third FWM processes, which leads to their good noise balance, thus the performance of the PC with quantum correlation between beams 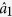 and 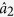 is not as sensitive to the *G*_2_ as the one in the asymmetrical cascaded FWM scheme, where only beam 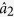 experiences the amplification, leading to noise unbalance. The study of 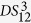 presented above is actually the question of how to preserve the quantum correlation between beams 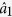 and 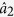 under the introduction of two FWMs which bring the deterioration effect to the quantum correlation by the quantum amplification of both the beams (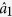, 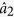). The results shown in Figs 5(b) and 6(b) actually shows the boundary for the values of *G*_2_ below which the quantum correlation can always be preserved as the value of *G*_1_ increases. More interestingly, any value of *G*_2_ more than 2 will eliminate the possibility of the existence of PC with quantum correlation between beams 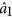 and 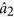 regardless of the value of *G*_1_.

PC between 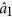 and 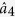 (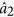 and 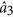) can be quantified by


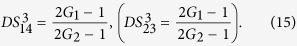


Eq. (15) is similar to the case of Eq. (10). The region in which 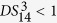 (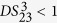) is shown in magenta denoted as ((1, 4), (2, 3)) in Fig. 5(b) and the contour plot is shown in Fig. 6(c). Therefore, beams 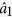 (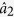) and 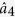 (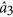) are quantum correlated within the magenta region (*G*_1_ < *G*_2_) in Fig. 5(b).

PC between 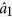 and 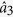 (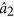 and 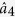) can be quantified by


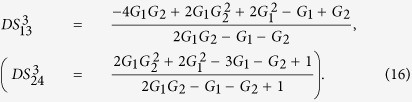


**Figure 7:**
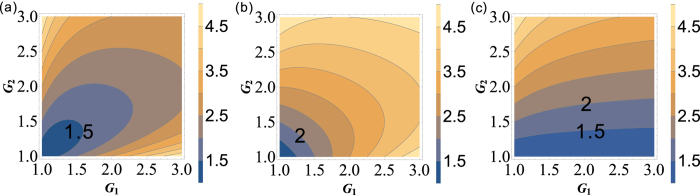
The contour plot of (**a**) 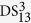; (**b**) 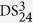 and (**c**) 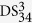.

Eq. (16) is always larger than 1 for any value of *G*_1_, *G*_2_ > 1 as shown in the contour plot of 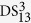 and 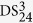 in Fig. 7(a) and (b). In this sense, there isn’t any quantum correlation between beams 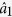 and 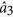 (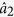 and 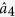) since 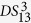 (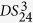) is always larger than 1 for any value of *G*_1_, *G*_2_ > 1. The absence of 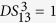 (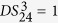) here compared with the red line in Fig. 1(b) is due to that both the beams 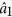 and 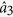 (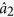 and 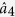) are amplified by the second and the third FWM processes independently.

PC between 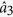 and 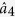 can be quantified by


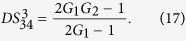


The PC with quantum correlation between beams 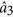 and 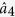 will be absent because *G*_2_ is always more than or equal to 1. This can be easily found if one looks at the functional form of Eq. (17). Its contour plot is shown in Fig. 7(c) in which the value of all the region is more than 1 for any G_1_, *G*_2_ > 1. As discussed above, for this symmetric cascaded scheme, there are three possible PCs with quantum correlation, namely 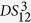, 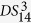 and 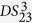. In addition, the existence of quantum correlation between beams 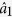 and 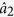 eliminates the possibility of the one between beams 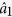 (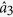) and 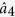 (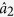). In other words, beam 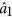 (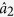) can’t be simultaneously quantum correlated with beam 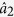 (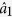) and 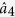 (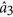). These effects in this symmetric scheme are similar to the above mentioned repulsion effect of quantum correlation between the PCs in the asymmetrical cascaded FWM process. Firstly, for the PC between beams 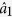 and 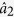, clearly, cell_1_ will provide the correlation between them while cell_2_ and cell_3_ will destroy their quantum correlation by adding extra vacuum noise, thus cell_1_, cell_2_ (cell_3_) can be viewed as the correlation mechanism provider and decorrelation mechanism provider respectively, thus the larger *G*_1_ and smaller *G*_2_ are preferred for the PC between beams 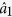 and 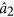. Secondly, for the case of the PC between 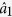 and 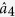 (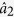 and 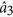), cell_1_ will generate two thermal states which will destroy their quantum correlation by adding extra vacuum noise into the system while the cell_3_ (cell_2_) will make them quantum correlated through the FWM processes. In this case, cell_1_, cell_3_ (cell_2_) can be viewed as the decorrelation mechanism provider and correlation mechanism provider respectively, thus the smaller G_1_ and larger G_2_ are preferred for the PC between beams 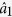 and 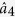 (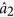 and 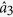). Finally, the complete opposite dependence of the PC between beams 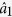 and 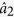 and the PC between beams 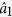 and 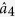 (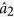 and 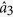) on the gains results in the repulsion effect between the PCs of certain pairs. In the blank region of Fig. 5(b), all of the PCs have no quantum correlation since 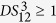, 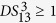, 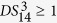, 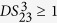, 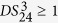 and 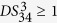, however, the quantum correlation between the quadruple beams is still present.

**Figure 8:**
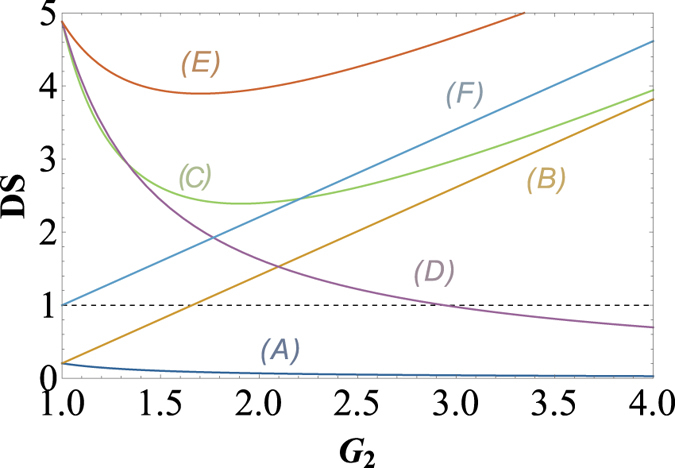
The dependence of (*A*) 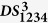; (*B*) 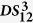; (*C*) 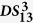; (*D*) 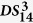 and 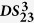; (*E*) 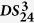 and (*F*) 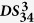 on the gain G_2_ when G_1_ = 2.94 (cell_1_ gain in the experiment). The black dashed line: SQL.

Here we also give a summary of the theoretical predictions of Figs 5(b), 6 and 7. We plot the dependence of the (*A*) 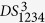; (*B*) 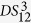; (*C*) 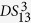; (*D*) 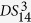 and 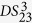; (*E*) 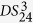 and (*F*) 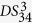 on the gain G_2_ when G_1_ = 2.94 (cell_1_ gain in the experiment) in Fig. 8. 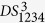 (trace *A*) can be enhanced with the increasing of *G*_2_ which is consistent with Fig. 6(a), the value of 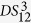 (trace *B*) will be larger than 1 as long as G_2_ > 1.67 which is consistent with the boundary (*G*_2_ = 2 − 1/*G*_1_) in Fig. 5(b), the value of 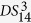 and 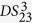 (trace *D*) will be smaller than 1 as long as G_2_ > 2.9 which is consistent with Figs 5(b) and 6(c). In addition, 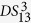 (trace *C*), 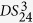 (trace *E*) and 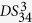 (trace *F*) are also consistent with Fig. 7(a–c) respectively.

**Figure 9:**
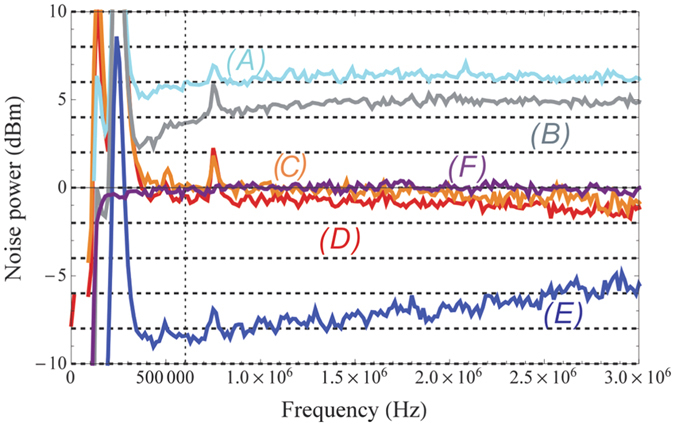
Experimentally measured (*A*) 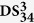; (*B*) 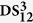; (*C*) 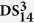; (*D*) 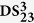; (*E*) 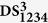 and (*F*) the corresponding SQLs of the traces *A* ~ *E* in the symmetrical cascaded scheme. The vertical dashed line: 0.6 MHz.

We have also applied these theoretical predictions to the experimental results of the symmetrical cascaded scheme as shown in Fig. 9, the traces *A, B, C, D* and *E* are the measured DSs between 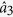 and 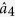, 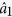 and 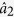, 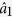 and 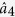, 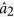 and 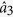 and the quadruple beams respectively, the trace F is the corresponding normalized SQLs for traces *A* ~ *E* (See the methods). The experimental results show 10Log(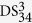) = 5.9 ± 0.3 dB, 10Log(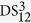) = 3.8 ± 0.6 dB, 10Log(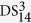) = 0.1 ± 0.4 dB, 10Log(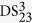) = −0.2 ± 0.7 dB and 10Log(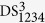) = −8.2 ± 0.5 dB at 0.6 MHz. For the experimental gains G_1_ ≈ 2.94 and G_2_ ≈ 2.85, the theoretical predictions give 10Log(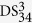) = 5.1 dB, 10Log(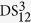) = 3.9 dB, 10Log(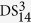) = 0.2 dB, 10Log(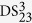) = 0.2 dB and 10Log(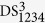) = −13.6 dB. As we can see, although these theoretical predictions do not perfectly agree with the experimental results at 0.6 MHz, they still give a rough estimation of the relationship between the obtained experimental noise power traces.

## Discussion

The PCs existed in the asymmetrical cascaded scheme and symmetrical cascaded scheme are both studied. We found that the symmetrical cascaded scheme has the following distinctions compared with the asymmetrical cascaded scheme: (1) Quantum enhancement. The DS of the quadruple beams in the symmetrical cascaded scheme (Eq. 13) has quantum enhancement compared with the one of the triple beams in the asymmetrical cascaded scheme (Eq. 7) with the same gains; (2) Boundary effect. The boundary of the PC with quantum correlation between beams 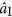 and 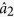 in the asymmetrical cascaded FWM scheme shown in Fig. 1(b) is obviously different from the one of the symmetrical cascaded FWM scheme shown in Fig. 5(b). This is because here beams 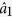 and 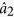 from the first FWM process experience the same amount of amplification in the second and third FWM processes, which leads to their good noise balance, thus the performance of the PC with quantum correlation between beams 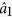 and 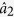 is not as sensitive to the *G*_2_ as the one in the asymmetrical cascaded FWM scheme, where only beam 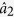 experiences the amplification, leading to noise unbalance. (3) SQL Approaching. The PC between beams 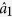 and 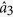 in the asymmetrical cascaded FWM scheme is clearly different from the one in the symmetrical cascaded FWM scheme. The PC between beams 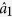 and 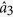 in the asymmetrical cascaded FWM scheme can approach its corresponding SQL (see the trace *C* in Fig. 3), while the PC between beams 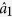 and 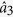 in the symmetrical cascaded FWM scheme is always much higher than its corresponding SQL (see the trace *C* in Fig. 8). This is because in the asymmetrical cascaded FWM scheme only one beam 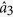 is amplified by the second FWM process, while in the symmetrical cascaded FWM scheme both of the beams 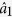 and 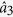 are amplified by the second and third FWM processes independently.

In summary, we have theoretically characterized the performance of the PCs from the multiple quantum correlated beams and analyzed the dependence of all the PCs on the system intensity gains based on two different cascaded FWM processes. For both cases, we have theoretically predicted the so called repulsion effect of quantum correlation between the PCs of the cascaded systems. Our results presented here can be applied to the classification and application of the quantum states generated from the cascaded FWM processes.

## Methods

### Experimental measurements of PCs

The output beams 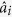 (*i* = 1, 2, 3, and 4) from the cascaded FWM processes are sent to the photodetectors and their noise power values *N*_*i*_ (*i* = 1, 2, 3, and 4) are measured. One beam is subtracted from the other beam in the pairwise beams and thus the intensity-difference squeezing shared by the pairwise beams is measured. In addition, the SQL of the measured pairwise beams can be measured in this way by using a beam in a coherent state with a power equal to the total power of the measured pairwise beams impinging on the photodetectors. We then split it with a 50/50 beamsplitter, direct the obtained beams into two photodetectors. and record the noise power of the differential photocurrent. This balanced detection system makes it possible to cancel all the sources of classical noise and obtain a measure of the SQL.

## Additional Information

**How to cite this article**: Wang, H. *et al*. Characterization of Pairwise Correlations from Multiple Quantum Correlated Beams Generated from Cascaded Four-Wave Mixing Processes. *Sci. Rep.* **7**, 40410; doi: 10.1038/srep40410 (2017).

**Publisher's note:** Springer Nature remains neutral with regard to jurisdictional claims in published maps and institutional affiliations.
